# Regulatory Network of Leaf Senescence in *Arabidopsis*: The Roles of Transcription Factors and microRNAs

**DOI:** 10.3390/ijms27083584

**Published:** 2026-04-17

**Authors:** Yu Zhai, Wenguang Qiao, Wen Yang, Xunyan Liu

**Affiliations:** College of Life and Environmental Sciences, Hangzhou Normal University, Hangzhou 311121, China; 18245931368@163.com (Y.Z.); qiao991232223@163.com (W.Q.); sheep177250@163.com (W.Y.)

**Keywords:** *Arabidopsis*, dark-induced senescence, leaf senescence, miRNAs, post-transcriptional regulation, transcriptional regulation, transcription factors

## Abstract

Leaf senescence, the final developmental stage of a leaf, is a highly regulated process that is vital for the recycling of nutrients and the maintenance of plant fitness. Its control operates at multiple levels, including chromatin remodeling, transcription, post-transcriptional regulation, translation, and post-translational modifications. This review summarizes recent advances in understanding the roles of key transcription factor (TF) families—WRKY, NAC, and MYB—in modulating leaf senescence in *Arabidopsis thaliana*. We detail how these TFs integrate internal and external signals to regulate senescence-associated genes (SAGs). In addition, we explore the pivotal role of microRNAs (miRNAs) in post-transcriptional control of senescence, focusing on their regulation of these TF families. In conjunction with the transcriptome data of *Arabidopsis* miRNAs under conditions of dark-induced senescence, we also highlight several novel senescence-associated miRNAs. Integrating transcriptional and post-transcriptional perspectives, this review presents an updated regulatory network for leaf senescence and discusses potential applications for manipulating senescence in crops to improve yield and quality.

## 1. Introduction

Leaf senescence, the final stage of leaf development, is a highly regulated process crucial for nutrient recycling and overall plant fitness. As the primary organ for photosynthesis, the senescence of leaves profoundly impacts plant growth, development, and reproduction [1]. From the perspective of plant life cycles, leaf senescence is not only an inevitable stage in natural development, but also an adaptive response of plants to environmental changes [2]. During the process of senescence, plants actively remobilize nutrients, such as nitrogen (N), phosphorus, and potassium, from their leaves and redistribute them to other vigorously growing tissues and organs, a process that is essential for plant survival and reproduction [3]. For example, in the later stages of crop growth, nutrients released from leaf senescence can provide sufficient nutrients for seed development and maturation, which directly affects crop yield and quality [4]. However, the regulatory mechanism of leaf senescence is extremely complex, involving the synergistic effects of a variety of internal and external factors, including phytohormones, environmental signals, TFs, and epigenetic modifications, among others [5,6].

TFs, which specifically bind DNA cis-acting elements and regulate gene transcription, play an important role in the regulatory network of plant gene expression [7]. During leaf senescence, TFs regulate the initiation and progression of leaf senescence by binding to the promoter regions of *SAGs* and modulating the expression of these genes [8]. In recent years, advances in molecular biology have led to the identification and functional characterization of numerous TF families involved in leaf senescence, such as, the family named for its conserved WRKYGQK motif (WRKY), the family named for the three founding members NAM, ATAF1/2, and CUC2 (NAC), and the family named after the v-myb avian myeloblastosis viral oncogene homolog (MYB), and others [9]. While transcriptional regulation by TFs has been extensively studied, the role of post-transcriptional regulation in leaf senescence remains to be further explored. Most existing studies have focused on individual members of these TF families, and there is a lack of a unified understanding of their coordinated roles in leaf senescence. In addition to transcriptional regulation, post-transcriptional regulation plays an indispensable and important role, especially miRNAs-mediated regulatory mechanisms [10]. miRNAs are a class of endogenous non-coding RNAs of approximately 20–24 nt in length, that guide the cleavage or translational repression of target mRNAs through base complementarity [11]. In plants, miRNAs are involved in the regulation of numerous biological processes, such as growth and development, response to adversity, and senescence [12]. During leaf senescence, miRNAs can indirectly affect the process of leaf senescence by regulating the expression of senescence-related TFs [13].

Although previous reviews have summarized significant progress regarding various TF families involved in leaf senescence, there remains a lack of systematic reviews that integrate the transcriptional regulation of WRKY, NAC and MYB TFs with their post-transcriptional regulation by miRNAs. In particular, the miRNA-mediated post-transcriptional regulatory layer in *Arabidopsis*, especially its interactions with senescence-associated TF modules, has not yet been comprehensively summarized. Our transcriptomic studies of dark-induced senescence have identified several novel senescence-associated miRNAs. However, their potential links with established TF networks remain under-explored. Therefore, this review aims to synthesize current knowledge regarding the WRKY, NAC, and MYB regulatory modules, highlight newly identified senescence-related miRNAs under dark-induced conditions, and integrate these findings into an updated regulatory framework from both transcriptional and post-transcriptional perspectives to bridge current knowledge gaps and serve as a reference for future crop improvement strategies. Such insights may guide efforts to manipulate this process in crops, and through molecular breeding, could ultimately contribute to enhanced agricultural productivity.

## 2. Senescence Regulation by a Family of TFs

### 2.1. WRKY TF Family Regulates Leaf Senescence

WRKY TFs are extensively involved in regulating the expression of senescence-related genes in *Arabidopsis* leaves. Numerous members, including WRKY1, WRKY6, WRKY18, WRKY22, WRKY25, WRKY26, WRKY30, WRKY41, WRKY42, WRKY45, WRKY46, WRKY47, WRKY53, WRKY54/70, WRKY55, WRKY57, WRKY71, WRKY75 have been implicated in this process [14,15,16,17,18,19,20,21,22,23,24,25,26,27,28,29]. Most WRKY TFs act as positive regulators of leaf senescence, whilst a small number act as negative regulators or regulate the process in complex ways. Typically, most WRKY TFs respond to salicylic acid (SA) signaling. However, the senescence regulatory network is far more complex than this. Some TFs respond not only to SA but also to multiple plant hormones. For example, the WRKY75 TF also responds to Jasmonic Acid (JA), Ethylene (ET) and Abscisic Acid (ABA). These TFs are summarized in Table 1.

Studies indicate that WRKY TFs regulate senescence through two primary mechanisms [32]. For instance, some members, such as WRKY1 and WRKY47, regulate target gene expression directly by binding to the W-box elements in their promoter regions. WRKY1 is a key regulator of monocarpic senescence in *Arabidopsis thaliana*. Its expression is induced by a variety of signals, including age, SA, and N deficiency. Studies have shown that WRKY1 promotes the initiation of flowering by directly repressing the expression of the flowering repressor gene *FLOWERING LOCUS C* (*FLC*), and activates the key genes for SA synthesis, *SA INDUCTION DEFICIENT 2* (*SID2*) and *AVRPPHB SUSCEPTIBLE 3* (*PBS3*), which in turn drives the leaf senescence process. In addition, WRKY1 also directly up-regulated N assimilation genes and N transporter genes to promote N redistribution from leaves to seeds, and it systematically regulated monocarpic senescence by integrating the environmental signals with internal developmental signals and synergistically regulating the three major processes of flowering, leaf senescence and N reutilization [14]. WRKY47, a positive regulator of leaf senescence, specifically binds to the W-box (TTGACT/C) cis-acting elements in the promoters of the programmed cell death (PCD) genes *BIFUNCTIONAL NUCLEASE 1* (*BFN1*) and *METACASPASE 6* (*MC6*) through its conserved WRKY domain, directly activates these two PCD genes and up-regulates the expression of *SAGs* (*SAG12* and *SAG13*), which ultimately promotes age-dependent leaf senescence. In this study, a key molecular mechanism for the association between leaf senescence and programmed cell death was identified [25]. In addition, WRKY41 was identified as a novel factor in the regulation of leaf senescence in *Arabidopsis thaliana* via single-cell nuclear transcriptome technology. It is highly expressed in senescent chloroplasts and vascular cells, and may regulate senescence through interactions with members of the NAC family and autophagy genes, and thus adds a new core target in the transcriptional regulation network of leaf senescence [20]. Other members exert their function within complex protein regulatory network by interacting with other WRKY TFs to achieve signal integration and multilevel regulation. WRKY22 not only participates in regulating the expression of senescence-related genes *SAG12*, *SAG20*, and the *SENESCENCE-INDUCED RECEPTOR-LIKE KINASE* (*SIRK*), but also plays a central role in the transcriptional regulatory network of leaf senescence by forming a network with family members such as WRKY6, WRKY53, and WRKY70, collectively integrating senescence signals [17]. WRKY30 can independently interact with the positive regulator WRKY53 and the negative regulators WRKY54/WRKY70 in a heterologous system. This finding further highlights the functional redundancy among members of the WRKY family in the regulation of leaf senescence. For example, WRKY54 and WRKY70 act as partially redundant negative regulators. The *wrky70* single mutant exhibits a mild premature senescence phenotype, whereas the *wrky54* single mutant shows no obvious phenotype, whilst the *wrky54 wrky70* double mutant exhibits a significantly exacerbated premature senescence phenotype [19]. WRKY25 serves as a key activator in the leaf senescence regulatory network. Its N-terminal domain is the core region responsible for activating *WRKY53* expression and is indispensable for forming heterodimers with the repressor WRKY18. Conversely, the C-terminal domain limits its activity to prevent excessive *WRKY53* expression. The heterodimer formed by WRKY25 and WRKY18 regulates *WRKY53* expression via redox-sensitive covalent NOS bridges, constructing a finely-tuned regulatory WRKY18/WRKY25/WRKY53 subnetwork [16]. Consequently, this regulatory network, comprising multiple WRKY TFs, is capable not only of compensating for the effects of functional loss in individual members, but also of finely tuning the senescence process through differences in spatiotemporal expression, protein interactions and the intensity of transcriptional regulation. It is thus evident that the functional redundancy and synergistic interactions within the WRKY family may constitute the key foundation for the stability and flexibility of the leaf senescence network.

In conclusion, the WRKY TF family demonstrates its core regulatory value in the regulation of leaf senescence. It is recommended that future studies can further explore their functions in different plant species and under different environmental stresses. In addition, it would be worthwhile to investigate their potential application in crop genetic improvement to delay senescence and improve N utilization efficiency and yield. This would provide new strategies for the sustainable development of modern agriculture.

### 2.2. NAC TF Family Regulates Leaf Senescence

The NAC family is one of the largest families of TFs unique to plants. Members of the NAC family are characterized by the presence of highly conserved N-terminal DNA-binding domains and a diverse array of C-terminal structural domains [33]. Numerous NAC TFs are key regulators of leaf senescence, including ANAC029 (AtNAP), ANAC059/ORGAN SIZE-LIKE PROTEIN (ORS1), ANAC042/JUNGBRUNNEN1 (JUB1), ANAC016, ANAC102/019/055/081/032, ANAC002/ATBS1-INTERACTING FACTOR1 (ATAF1), ANAC072 (RD26), ANAC032, ANAC046, ANAC017, ANAC082, ANAC090, ANAC083/VND-INTERACTING2 (VNI2), ANAC092/ORESARA1 (ORE1), ANAC087, ANAC075 [34,35,36,37,38,39,40,41,42,43,44,45,46,47]. We have summarized the role of NAC TFs in regulating leaf senescence in Table 2.

NAC TFs often integrate multiple signals to directly regulate senescence. ANAC032 integrates multiple senescence signals and acts upstream of several regulatory pathways. ANAC032 integrates a number of physiological processes including age signaling, dark treatment, oxidative stress, salt osmotic stress and hormone signaling, thereby promoting leaf senescence in *Arabidopsis*. The accumulation of reactive oxygen species (ROS) disrupts intracellular structures, leading to chlorophyll degradation and regulating the expression of downstream *SAGs*, such as *SAG12*, *SAG113*, and *SMALL AUXIN UP RNA 36* (*SAUR36*). *ANAC032* overexpression plants exhibit premature leaf senescence and accelerated chlorophyll degradation, whereas functionally deficient plants exhibit delayed senescence [41]. ATAF1 is also a key regulatory factor that directly responds to ABA and H_2_O_2_ signals and plays an upstream regulatory role in hormone signaling. ATAF1 is an ABA and H_2_O_2_-inducible NAC TF that regulates leaf senescence by directly binding to and upregulating *ORE1* and *NINE-CIS-EPOXYCAROTENOID DIOXYGENASE 3* (*NCED3*) (key ABA synthesis gene), while simultaneously suppressing *GOLDEN2-LIKE 1* (*GLK1*) (chloroplast maintenance factor) expression. Furthermore, ATAF1 directly activates *ABC TRANSPORTER G FAMILY MEMBER 40* (*ABCG40*) (ABA transporter protein gene), potentially enhancing intracellular ABA accumulation, thereby playing a central regulatory role in ABA and H_2_O_2_-mediated senescence signaling pathways [39]. The *VNI2* gene is influenced by abiotic factors, specifically the phytohormone ABA and salt stress. Under salt stress, VNI2 acts as a transcriptional activator, directly binding to the promoter of the *COLD-REGULATED (COR) and RESPONSIVE TO DEHYDRATION (RD)* genes and thereby activating their expression. The present study posits that the overexpression of the *COR* or *RD* genes delayed leaf senescence, suggesting that VNI2 integrates ABA-mediated salt stress signaling into the regulation of leaf senescence through the regulation of these genes to prolong leaf longevity [44]. By contrast, ANAC072 is located downstream of the senescence signaling pathway. ANAC072 has been demonstrated to promote chlorophyll catabolism through a direct binding process to the promoter of *NON-YELLOWING 1 (NYE1)*, a key gene for chlorophyll degradation. This process serves to regulate both natural and dark-induced leaf senescence. Loss-of-function mutants of *anac072* exhibited significantly delayed leaf degreening under both natural and dark conditions, and detected higher chlorophyll levels in both natural and dark conditions. In contrast, the overexpression mutant exhibited premature yellowing and accelerated senescence under identical conditions [40].

These seven genes (*ANAC019/055/072/002/081/102/032*) are members of the A subfamily of stress-responsive NAC (SNAC-A) subfamily, which collectively function as key positive regulators in ABA-induced senescence. The SNAC-A septuple mutants significantly delayed ABA-induced leaf senescence, as evidenced by the reduction in chlorophyll degradation, the maintenance of intact chloroplast structure, and the suppression of the expression of senescence-related genes, such as *SAG12*, *SAG26*, and *EARLY RESPONSIVE TO DEHYDRATION 1 (ERD1)*. This suggests that the SNAC-A TF plays a key role in ABA-induced leaf senescence. Of note, the single-mutant forms of these SNAC-A members do not exhibit any obvious senescence phenotype, suggesting that there is significant functional redundancy within this subfamily, enabling ABA-induced senescence signaling to proceed normally even in the event of loss of function in a single gene [38]. Conversely, JUB1, ANAC017, ANAC082 and ANAC090 act as negative regulators in the regulation of senescence. JUB1 negatively regulates senescence by activating antioxidant defense pathways and reducing cellular H_2_O_2_ levels in *Arabidopsis thaliana*. The overexpression of *JUB1* has been demonstrated to significantly retard leaf senescence. This retardation is evidenced by a slowing down of chlorophyll degradation and the down-regulation of the expression of senescence-related genes. In contrast, the *JUB1* loss-of-function mutant, *jub1-1*, exhibited a premature senescence phenotype, accompanied by increased H_2_O_2_ accumulation [36]. *ANAC017*, *ANAC082*, and *ANAC090* are synergistically expressed during the presenescent stage of *Arabidopsis* leaves, constituting a cooperative negative regulatory module of senescence [43]. The function of this module is to retard senescence by inhibiting the SA and ROS signaling pathways. Specifically, ANAC090 has been shown to directly bind to and repress the promoters of SA synthesis and signaling genes, thereby reducing SA accumulation. Meanwhile, ANAC017 exerts a negative regulatory influence on genes implicated in ROS homeostasis, thereby averting premature ROS accumulation. The regulatory functions of these three factors are enhanced through the formation of homodimers and heterodimers. Genetic evidence further supports their functional redundancy: while single mutants of *anac017*, *anac082*, or *anac090* show no apparent senescence defects, combining mutations in two or three of these genes leads to accelerated leaf senescence. This multi-layered redundancy ensures that the loss of any single member does not compromise the overall suppression of senescence, providing robustness to the regulatory network under fluctuating environmental conditions [43].

In summary, the NAC TF family plays a central role in regulating leaf senescence. Although considerable research has been conducted on the identification of individual members, several key questions remain unresolved. These include the hierarchical relationships among NAC members, the molecular mechanisms underlying functional redundancy and specificity, the mechanisms by which multiple signaling pathways are integrated, and the degree of conservation of *Arabidopsis* in crops. Future research that integrates multi-omics approaches, protein interaction analysis and crop functional genomics will be crucial in translating NAC-mediated senescence regulation into agricultural improvements.

### 2.3. The MYB and Other TF Families Regulate Leaf Senescence

The MYB TF family is one of the most widely distributed and functionally diverse families of TFs in plants and is involved in a variety of biological processes in *Arabidopsis* [48]. Several MYB factors regulate leaf senescence positively and negatively. Positive regulatory factors accelerate senescence by integrating stress signals. *AtMYBL* is an R-R-type MYB-like TF, the expression of which is significantly up-regulated with leaf senescence progression. It has been demonstrated that this factor can be induced by ABA and salt stress [49]. *MYBH* exhibited a substantial enhancement in expression in senescent leaves and dark-treated leaves [50]. AtMYB2 regulates senescence throughout the entire plant in *Arabidopsis* by inhibiting Cytokinin (CTK) synthesis to prevent axillary bud emergence during late development [51]. GLABRA 1 (GL1) is a MYB TF belonging to the R2R3 subfamily, which upregulates dark-induced senescence. In *gl1* mutants, the senescence process is markedly slowed, photosynthetic gene expression is downregulated, and the induction of senescence marker genes is attenuated [52]. Negative regulators, in contrast, often function by suppressing hormone biosynthesis or signaling. AtMYBR1 has been identified as a negative regulator of the ABA signaling pathway. Overexpressing of *MYBR1* significantly delays leaf senescence and chlorophyll degradation [53]. In contrast, *MYB59* is predominantly expressed during the initial phase of leaf senescence and functions by directly repressing the transcription of the senescence-related genes *SAG18*, the SA synthesis genes *ISOCHORISMATE SYNTHASE 1* (*ICS1*) and *PHE AMMONIA LYASE 2* (*PAL2*), and the JA synthesis gene *LIPOXYGENASE 2* (*LOX2)* [54]. The expression of *MYB47* was found to be elevated in the course of leaf senescence. Overexpression of *MYB47* was found to significantly delay leaf senescence in *Arabidopsis*, whereas the converse was true in the *myb47* mutant. *MYB47* has been shown to bind directly to the promoters of the JA catabolic genes *CYP94B3* and *CYP94C1*, thereby activating their expression and reducing the level of endogenous JA in plants [55].

In addition to the WRKY, NAC and MYB families, which are well-characterized, recent studies have also identified several other TF families that have been shown to be key regulators of leaf senescence. They exert their effects by regulating growth and development or by integrating hormonal signaling pathways. ORESARA15 (ORE15) regulates leaf growth by promoting cell proliferation, thereby influencing the onset of senescence. ORE15, as a plant A/T-enriched sequence-binding zinc-containing protein (PLATZ) family TF, regulates leaf growth by promoting cell proliferation. Overexpressing *ORE15* significantly increases both the rate and duration of cell proliferation, accompanied by upregulation of cell cycle-related genes, growth regulators *GRF-INTERACTING FACTOR 1* (*GIF1*)/*ANGUSTIFOLIA 3* (*AN3*) and *GROWTH-REGULATING FACTOR 5* (*GRF5*), and suppression of *miR396a* expression [56]. *CDF4* promotes senescence by acting as a regulatory node in the ABA and ROS pathway. CDF4, a member of the DNA-binding with one finger (DOF) family of TFs, directly binds to the promoters of *NCED2* and *NCED3*, thereby upregulating their expression and elevating endogenous ABA levels. Additionally, *CDF4* represses the transcription of the *CATALASE 2* (*CAT2*), which suppresses H_2_O_2_ scavenging capacity and promotes ROS accumulation [57]. Furthermore, some TFs are directly involved in the cascade amplification of senescence signals. AtFOX1, a member of the forkhead box (FOX) family of TF, has been shown to positively regulate leaf senescence, and loss-of-function mutants of this gene exhibit delayed leaf senescence, while overexpression lines display a premature senescence phenotype [58]. The expression of RAV1, a member of the RAV family of TF, has been observed to increase during the process of late leaf maturation, reach a peak in early senescence, and can be induced by 1-aminocyclopropane-1-carboxylic acid (ACC) and methyl jasmonate (MeJA) [59].

In summary, a comprehensive overview of the functions of the MYB family and other families in regulating leaf senescence has been provided in Table 3. It is becoming evident that an increasing number of other families of TFs are involved in the leaf senescence process. Although the functional redundancy of age-related MYB TFs has not yet been extensively studied, their regulatory patterns show significant overlap with those of WRKY and NAC TFs. Some MYB TFs are involved in age, hormone or stress response pathways, and influence chlorophyll degradation, reactive oxygen species homeostasis, and the expression of age-related genes. However, the current understanding of the roles these TFs play in the regulation of senescence remains limited. How these TFs interact with one another and with NAC and WRKY TFs, the mechanisms by which they exert their effects, and the extent to which their functions are redundant or specialized across different tissues and environmental conditions remain largely unclear. Consequently, future research should focus on utilizing genetic and systems biology approaches to elucidate the hierarchical relationships between the NAC, MYB, WRKY and other TF families. It should also aim to decipher how hormonal, ROS and developmental signals are integrated within specific TFs. Furthermore, the role of these regulatory nodes in crop production should be validated; by modulating the senescence processes controlled by TFs, it may be possible to enhance crop yield, stress tolerance and quality.

## 3. Regulation of Leaf Senescence-Related TFs by miRNAs

Increasing evidence suggests that miRNAs do not act alone in influencing the leaf senescence process, but rather play a pivotal role within a multi-layered transcriptional regulatory network. Within this network reside several key TFs closely associated with leaf senescence, particularly the NAC and WRKY families, alongside proteins such as TEOSINTE BRANCHED/CYCLOIDEA/PCF (TCP), SQUAMOSA PROMOTER BINDING PROTEIN-LIKE (SPL), MYB and GROWTH-REGULATING FACTORS (GRF). It is precisely through these miRNA-TF complexes that plants establish interconnected pathways linking developmental stages, hormonal signaling, and environmental stress responses, ultimately achieving effective control over leaf senescence.

Among these regulatory modules, the miR164-NAC module is the most well-characterized. During leaf senescence, the ethylene signaling pathway component ETHYLENE INSENSITIVE2 (EIN2) mediates age-dependent downregulation of *miR164* expression. The reduction in *miR164* diminishes the cleavage inhibition of *ORE1* mRNA, leading to ORE1 protein accumulation. Studies indicate that ORE1 functions as a NAC family TF, further activating the expression of downstream *SAGs*, thereby positively regulating age-dependent cell death and the leaf senescence process [62]. Additionally, *HASTY* (*HST*) encodes an importin/exportin family protein that negatively regulates N-deprivation-induced leaf senescence in *Arabidopsis* by modulating miRNA dynamics. HST mutations accelerate senescence, whereas overexpression delays it. Under N-deprived conditions, interactions between HST and DICER-LIKE 1 (DCL1), RAS-RELATED NUCLEAR PROTEIN 1 (RAN1), and certain miRNAs diminish. This leads to reduced accumulation of senescence-related miRNAs such as *miR164a*, *miR169a*, and *miR781*, derepressing their target NAC TF genes *ORE1* and *NAC-LIKE*, *ACTIVATED BY AP3/PI* (*NAP*), which in turn promotes leaf senescence [63]. Beyond regulating the NAC core, miRNAs also modulate leaf senescence through pathways associated with WRKY factors and the JA signaling pathway. miR319 negatively regulates the TCP TFs by cleavage or transcriptional suppression. As transcriptional activators, TCP proteins bind to the *LOX2* promoter to upregulate its expression, leading to enhanced JA synthesis and the acceleration of leaf senescence. Under *miR319* overexpression conditions, TCP transcription is suppressed, reducing JA content and delaying both natural and dark-induced senescence [64]. Furthermore, *miR840*, which is highly expressed during the early stages of senescence, induces premature senescence by suppressing two negative regulators, *PENTATRICOPEPTIDE REPEAT (PPR)* and *WHIRLY3* (*WHY3*). Simultaneous inhibition of these two negative regulators activates multiple senescence-related regulatory factors such as *WRKY53* and *SAG12*, indicating that miR840 can target upstream negative regulators, release the inhibition on *WRKY53*, and subsequently activate its downstream senescence signaling network [65]. The third major regulatory layer concerns microRNA-mediated developmental stage transitions, a process that significantly influences the timing of senescence. miR396 accumulation decreases the levels of GRFs, which are negative regulators of leaf senescence. Therefore, miR396 promotes both natural and dark-induced leaf senescence [66]. However, miR156 has been demonstrated to maintain characteristics associated with juvenility and to delay the process of senescence by repressing the expression of SPL family TFs [67]. On the other hand, miR159 suppresses the target TF MYB33, thereby reducing its activation of ABA INSENSITIVE5 (ABI5). This weakens ABI5’s positive regulation of *miR156*, subsequently lowering *miR156* expression and alleviating its inhibition of SPL. Consequently, it promotes the transition from juvenile to adult states [68]. These studies collectively demonstrate that miRNAs exert significant post-transcriptional regulatory roles in leaf senescence, clustering around major modules centered on TFs. Within this model, NAC factors (e.g., ORE1 and NAP) bear primary responsibility for senescence, while WRKY-associated pathways amplify senescence signals. Concurrently, the SPL/TCP/MYB/GRF module governs developmental timing and hormone responsiveness. Consequently, miRNAs and TFs do not regulate these processes independently, but rather form an interactive network that includes signals from developmental cycles, stress, hormones and other pathways, collectively determining the senescence process.

**Figure 1:**
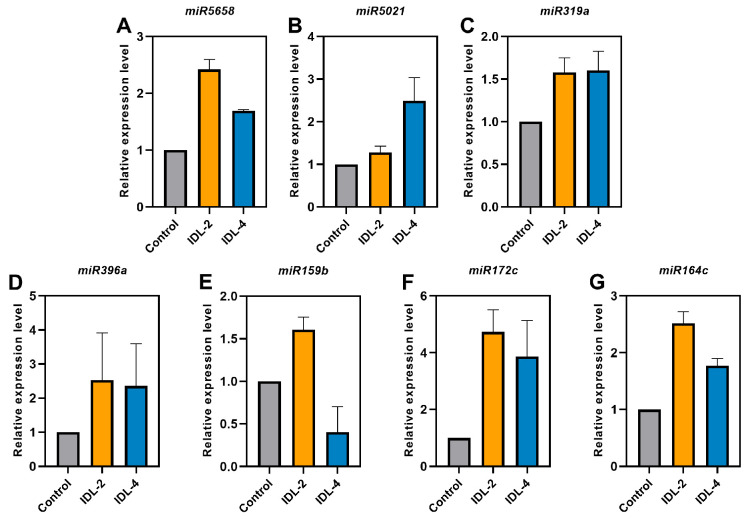
Expression levels of seven miRNAs quantified by real-time RT-PCR using stem-loop method under individually darkened leaf (IDL) induction. (**A**) *miR5658* (**B**) *miR5021* (**C**) *miR319a* (**D**) *miR396a* (**E**) *miR159b* (**F**) *miR172c* (**G**) *miR164c*. *UBQ6* served as an internal control, and error bars represent the standard deviation from three biological replicates.

Dark-induced leaf senescence is a well-established approach in the field of plant senescence research, and miRNAs, as key molecules in post-transcriptional regulation, have been shown to play an important role in the initiation and process regulation of dark-induced senescence [69]. Microarray technology analysis comprehensively revealed, for the first time, the expression characteristics and regulatory network of dark-induced senescence-associated miRNAs [69]. Based on the aforementioned microarray analysis results, we selected a subset of differentially expressed miRNAs and employed qRT-PCR to determine their expression levels, thereby validating the reliability of the microarray data. Among these genes, *miR319a*, *319c*, *miR159a*, *miR164a*, *164c*, *miR390a*, *miR408*, and *miR396a* have been identified as being involved in senescence [62,69,70,71,72,73,74]. The elevated expression levels of the *miR319a*, *miR164c*, and *miR396a* genes subsequent to dark treatment indicated that senescence was promoted under dark conditions, a finding that is consistent with the results previously reported in studies. Four additional miRNAs (*miR5658*, *miR5021*, *miR172c*, *miR159b*) have been identified by qRT-PCR as potentially implicated in leaf senescence under conditions of dark-induced stress (Figure 1).

**Figure 2:**
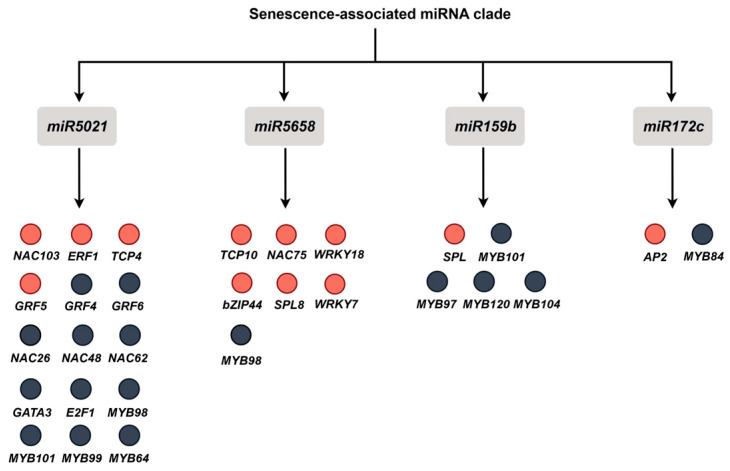
Predicted TF targets of the four miRNAs. Red circles indicate TFs for which there is existing evidence of involvement in plant growth, development, or leaf senescence, while black circles represent candidate factors for which no direct evidence has yet been reported. These results suggest that miR5021, miR5658, miR159b, and miR172c may form a regulatory network involved in leaf senescence by regulating known and putative senescence-related TFs.

Based on the qRT-PCR validation results, we further analyzed the regulatory roles of *miR5021*, *miR5658*, *miR159b*, and *miR172c* in darkness-induced leaf senescence. Predictions indicate that these miRNAs interact with TF families associated with multiple key developmental transitions and leaf senescence. Among the predicted targets of miR5021, NAC103 has been reported to positively regulate ABA signaling; since ABA is a key hormone that promotes senescence, NAC103 may be involved in the senescence process, although the mechanism remains to be verified. GRF5 promotes mesophyll cell expansion and chloroplast fission by enhancing auxin signaling [75]. Although its role in senescence has not been directly studied, its upstream repressor ARF2 has been shown to promote senescence [76]. Therefore, GRF5 may act as a negative regulator to delay senescence. ETHYLENE RESPONSE FACTOR 1 (ERF1) promotes seed germination by inhibiting ABA signaling and reducing ROS accumulation; given that both ABA and ROS promote senescence, ERF1 may also play a role in delaying senescence [77]. TCP4 accelerates senescence by activating *LOX2*, a key gene involved in JA synthesis, and inducing the expression of *WRKY53* [64]. The targets of miR5658 include *NAC75* and *WRKY18*, whose roles in senescence have been discussed previously. SPL8 inhibits GA signaling during the seedling stage, and since GA typically delays senescence, SPL8 may indirectly promote senescence [78]. Wheat *TaWRKY7* has been shown to positively regulate senescence, and based on functional conservation, *Arabidopsis AtWRKY7* may have a similar function [79]. TCP10 and TCP4 exhibit functional redundancy and may play a role in the positive regulation of senescence [80]. Furthermore, previous studies have shown that miR159 primarily participates in plant developmental processes by regulating the MYB family, while the miR156-SPL module plays a crucial role in phase transitions and senescence regulation [81]. Based on these findings, miR159b may directly target SPL TFs and may exert a novel regulatory mechanism in dark-induced leaf senescence. miR172c directly targets the APETALA2(AP2)/ERF TFs [82]. Taken together, these miRNAs may collectively participate in the regulation of dark-induced leaf senescence by targeting multiple key TFs (Figure 2).

**Figure 3:**
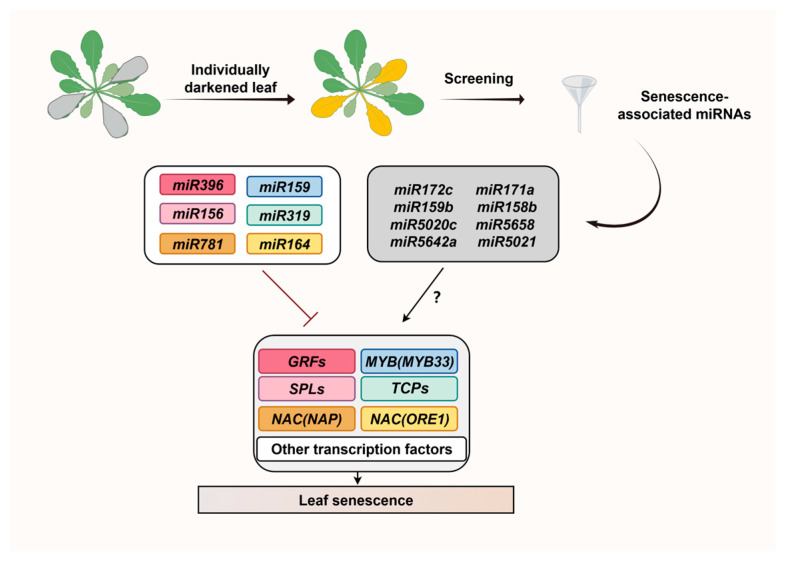
Following a dark-induced screening, two categories of senescence-associated miRNAs were identified: one comprises known senescence-associated miRNAs, which have been color-coded according to the TF families they target. Specifically, red miR396, pink miR156 and orange miR781 target the GRF, SPL and NAC (NAP) TF families, respectively, whilst blue miR159, cyan miR319 and yellow miR164 target the MYB (MYB33), TCPs and NAC (ORE1) TF families, respectively. These miRNAs directly participate in the leaf senescence process by inhibiting the expression of their corresponding target TFs. For example, the inhibition of ORE1 by miR164 is a classic senescence regulatory pathway. The second category comprises miR5020c, miR5642a, miR171a, and miR158b, which were successfully identified in previous dark-induced screens [69], along with the newly discovered senescence-associated miRNAs identified in this screen: miR5021, miR5658, miR172c, and miR159b marked with grey boxes. The TF families targeted by these miRNAs have not yet been identified. Further experimental validation will be pursued subsequently.

In summary, as shown in Figure 3, we have synthesized these findings and summarized the regulatory network of miRNAs associated with dark-induced senescence and their target TFs. Both known and newly identified miRNAs may collectively regulate leaf senescence by targeting multiple key TF families.

## 4. Conclusions and Future Perspectives

### 4.1. TFs and miRNA Regulatory Networks in Leaf Senescence

Leaf senescence in *Arabidopsis* is governed by a complex, multi-tiered regulatory network in which TFs and miRNAs act in concert. The majority of studies to date have focused on individual TF families or single regulatory levels, and there remains a lack of systematic analysis of the integration mechanisms between these levels. In this section, we integrate these findings to construct a regulatory network involving TFs and miRNAs, thus providing a novel perspective on the regulation of leaf senescence. This regulatory network consists of the following four components.

(i) Signal input. Leaf senescence is triggered by various endogenous and environmental signals. These include developmental age, darkness, plant hormones (e.g., ABA, SA, JA, ET), and ROS. These signals act on downstream regulatory factors through different pathways.
(ii) TF interactions. TF families, including WRKY, NAC, and MYB, have been identified as the core regulatory modules of this network. Various forms of interactions exist among them. For example, WRKY71 promotes leaf senescence by directly regulating the expression of the NAC family TF *ANAC092*/*ORE1*. Transcriptomic analysis indicates that WRKY71 also influences the expression of several other NAC family members, including *ANAC082*, *ANAC047*, *ANAC042*/*JUB1*, *ANAC053* and *NAC072*. Furthermore, WRKY71 regulates the expression of the MYB family TF *AtMYB2* [28]. It has recently been discovered that the WRKY10 TF positively regulates leaf senescence in rice, forming a regulatory module with the VQ MOTIF-CONTAINING PROTEIN 8 (VQ8). VQ8 negatively regulates the senescence process by inhibiting the transcriptional activity of WRKY10. Both WRKY10 and VQ8 interact directly with the basic leucine zipper (bZIP) family TFs ABA RESPONSIVE ELEMENT BINDING FACTOR1 (ABF1) and ABF2, and VQ8 also inhibits the transcriptional activation activity of ABF1/2. WRKY10 also regulates members of the NAC family (ONAC096, NAC2) to promote the expression of senescence-related genes [83].
(iii) miRNA-mediated post-transcriptional regulation. In addition to the direct regulation by the core set of TFs, miRNAs exert fine-tuned control over leaf senescence at the post-transcriptional level by targeting TF mRNAs. These miRNAs, together with their target TFs, form various regulatory modules, as systematically summarized in Section 3. For example, miR164-ORE1 forms a negative feedback loop [62], miR319-TCP influences JA synthesis [64], miR156-SPL and miR159-MYB33-ABI5 regulate developmental stage transitions [67,68], and miR840 achieves synergistic regulation by targeting PPR and WHY3 [65]. Thus, miRNAs and core TFs act in concert to form a multilevel regulatory network governing leaf senescence.
(iv) Downstream target genes. TFs and miRNA regulatory networks act in concert to regulate the expression of downstream genes, including those involved in chlorophyll degradation (*NYE1*, *SGR1*) [37,40], programmed cell death (*BFN1*, *MC6*) [25], and senescence markers (*SAG12*, *SAG13*) [22], thereby driving the onset of leaf senescence.

**Figure 4:**
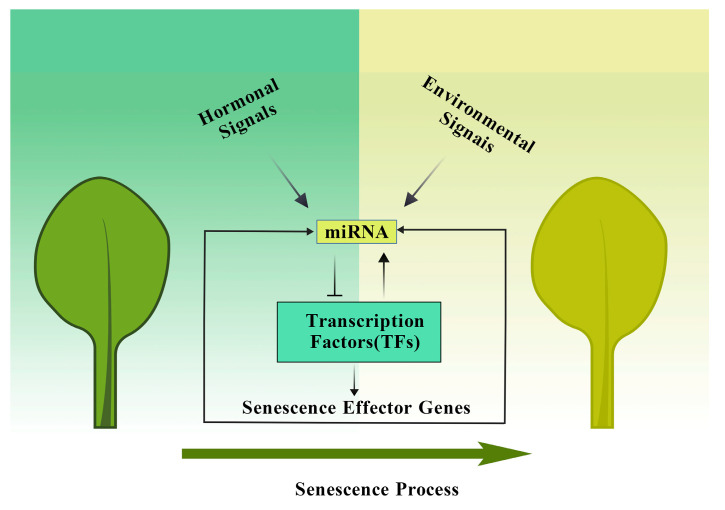
Schematic diagram of the signaling pathway of miRNA-TFs regulating leaf senescence. Environmental or hormonal signals regulate miRNA expression. miRNAs then act on TFs to further regulate senescence effector genes, ultimately completing the leaf senescence process. Created with https://biogdp.com/ [84].

TFs are key regulators of leaf senescence in *Arabidopsis*, responsible for integrating hormonal and environmental signals. miRNAs, on the other hand, primarily regulate the activity of TFs at the post-transcriptional level. Their complex interactions at multiple levels jointly coordinate the regulatory network governing leaf senescence. In this paper, we synthesize current knowledge on the roles of TFs (WRKY, NAC, MYB and others) and miRNAs in regulating leaf senescence in *Arabidopsis*. We also highlight newly identified miRNAs involved in this process by experimental validation (Figure 4), providing an updated view of the regulatory network. Despite the fact that current studies targeting the regulatory networks of miRNAs and TFs are mainly based on the model plant *Arabidopsis thaliana*, the core regulatory mechanisms are likely similar and highly relevant for crop improvement.

### 4.2. Conservatism and Translational Applications in Model Plants and Crops

#### 4.2.1. Cross-Family Interactions Among TFs

In recent years, biotechnological advances have led to the identification of an increasing number of TFs involved in leaf senescence. However, several key challenges remain in translating these findings into a comprehensive understanding of regulatory networks and agricultural applications. Drawing on the key findings of this review, we outline specific research directions to address these challenges.

One such direction involves exploring the interactions among different TF families in the context of leaf senescence. Although we have previously discussed the interactions among certain TF families in leaf senescence, systematic studies in this field remain relatively scarce. In contrast, studies on the interactions among different TF families have been reported in other crops or biological processes. For example, overexpression of the soybean (*Glycine max*) MYB TF *GmMYBLJ* significantly upregulates the expression of multiple WRKY genes (such as *AtWRKY30*, *AtWRKY45*, and *AtWRKY75*) and various senescence-related genes, while simultaneously promoting the accumulation of reactive oxygen species and accelerating leaf senescence [85]. In addition to these regulatory interactions, TFs can also form complexes through protein-protein interactions. For example, WRKY TFs can interact with MYB, bHLH, or WD40 to form MBW-WRKY (MBWW) protein complexes, which jointly regulate metabolic processes such as anthocyanin biosynthesis [86]. In potatoes (*Solanum tuberosum*), StMYB168 and StWRKY20 form a complex through direct protein-protein interactions, which enhances the transcriptional activation of the lignin synthesis genes *PHENYLALANINE AMMONIA LYASE 5 (PAL5)* and *CINNAMYL ALCOHOL DEHYDROGENASE 14 (CAD14)* by StMYB168, thereby promoting lignin accumulation during tuber wound healing [87]. Although these studies did not specifically address leaf senescence, they reveal that stable interaction modules can form between TFs from different families, providing important clues for elucidating the TF networks involved in leaf senescence.

#### 4.2.2. Conservation of *Arabidopsis* Regulatory Modules in Crops

In addition to interactions among TF families, several key components of the leaf senescence regulatory network in the model plant *Arabidopsis thaliana* are conserved in major crops. For example, NAC and WRKY TFs have been shown to be involved in the senescence process in rice and wheat, pointing to potential targets for genetic improvement in these important cereal crops. In rice (*Oryza sativa*), several NAC TFs are involved in the positive regulation of leaf senescence. In particular, the function of *OsNAC2* in rice is highly similar to that of its homologue (*ANAC092*/*ORE1*) reported in *Arabidopsis*, *OsNAC2* directly regulates chlorophyll degradation genes (*OsSGR* and *OsNYC3*), which is consistent with the regulatory mechanism of its homologue in *Arabidopsis* [88]. Furthermore, OsNAC103 also positively regulates leaf senescence by activating the ABA and JA signaling pathways and the expression of chlorophyll degradation genes [89]. Also, the activation mutant (*ps1-D*) of another NAC transcription factor, OsNAP, has been shown to accelerate leaf senescence and chlorophyll degradation. Conversely, RNAi-mediated knockdown of *OsNAP* significantly delays the process of rice senescence, thereby increasing yield by 6.3–10.3% [90]. In wheat (*Triticum aestivum*), several WRKY TFs have been identified as regulators of leaf senescence. Among these, TaWRKY42-B has been shown to be a positive regulator of leaf senescence, which induces leaf senescence by promoting JA accumulation through the activation of the JA synthesis gene *TaLOX3* [91]. Another wheat WRKY TF, TaWRKY40-D, exhibits functional activity in *Arabidopsis*, and its response patterns to JA and ABA are similar to the known functions of *WRKY57* and *WRKY75* in *Arabidopsis*, suggesting that the regulatory mechanisms are conserved between monocotyledons and dicotyledons [92]. In addition to WRKY TFs, NAC family TFs have also been reported to play critical roles in regulating wheat leaf senescence. For instance, wheat NAC5-1 has been shown to positively regulate leaf senescence. Loss-of-function mutants (*TILLING*) exhibited a significantly higher flag leaf chlorophyll content (SPAD) from 14 to 35 days after heading and a delayed time to 25% yellowing, showing a stay-green and delayed senescence phenotype. In contrast, overexpression lines exhibited a marked decrease in chlorophyll content from 21 to 35 days after heading and an earlier onset of senescence. Together, these gain- and loss-of-function genetic evidences demonstrate that NAC5-1 positively regulates leaf senescence in wheat by promoting chlorophyll degradation [93].

In maize (*Zea mays*), the NAC TFs ZmNAC132 and ZmNAC126 have both been identified as positive regulators of leaf senescence. ZmNAC132 directly activates the expression of *ZmNYE1*, a key gene involved in chlorophyll degradation [94]. ZmNAC126, on the other hand, not only directly activates several chlorophyll degradation genes (*ZmNYE1*, *ZmNYC1*, *ZmPAO*), but is itself directly regulated by ZmEIN3, a key factor in the ethylene signaling pathway [95]. This regulatory mechanism differs from the way in which EIN3 indirectly regulates *ORE1* via *miR164* in *Arabidopsis* [96], but both demonstrate the conservation of ethylene signaling in the regulation of leaf senescence. In Rapeseed (*Brassica napus*), WRKY TFs also play a significant role in leaf senescence. Among these, *Bna.A07.WRKY70* shares a highly conserved WRKY domain and phylogenetic relationship with *Arabidopsis WRKY70*. Functional complementation experiments indicate that the heterologous expression of *Bna.A07.WRKY70* in the *Arabidopsis wrky70* mutant restores its premature senescence phenotype and the expression levels of senescence marker genes (*AtSAG13*, *AtSEN1*), indicating that *Bna.A07.WRKY70* and *AtWRKY70* are functionally conserved in the regulation of leaf senescence, with both acting as negative regulators of leaf senescence [97]. Another WRKY TF, BnaA09.WRKY47, is induced under N-deficient conditions. By directly activating the key senescence gene *BnaC07.SGR1*, as well as the amino acid transporter gene *BnaA09.AAP1* and the nitrate transporter gene *BnaA02.NRT1.7*, it promotes the remobilization and transport of N within senescent leaves, thereby maintaining yield under low-N stress [98].

#### 4.2.3. Limitations of Conservation and Species-Specific Differences

However, whilst the aforementioned studies provide support for *Arabidopsis*-related senescence regulatory modules in rice, wheat, maize and oilseed rape, most are still based on sequence homology, heterologous expression or expression correlation analyses. Key comparative analyses between *Arabidopsis* and crops remain insufficient. In many cases, conservation is inferred primarily from homology rather than being directly validated by robust genetic and biochemical evidence. For instance, there remains a lack of systematic clarification regarding whether homologous genes retain the same downstream targets, regulatory hierarchies, and environmental response characteristics as their *Arabidopsis* counterparts. It is worth noting that even within the same conserved framework, different species may achieve similar senescence phenotypes through distinct molecular pathways. For example, in *Arabidopsis*, EIN3 indirectly regulates *ORE1* via *miR164* [96], whereas in maize, *ZmEIN3* directly binds to the *ZmNAC126* promoter [95], indicating that specific mechanisms exist across different species within conserved regulatory networks. Furthermore, differences in miRNA target sites, promoter structures and hormone interaction networks across species may further contribute to the differentiation of senescence regulatory outputs. Consequently, future research should not only continue to identify conserved genes but also systematically distinguish between conserved functions and lineage-specific adaptations through comparative genetics, cross-species complementation experiments, promoter analysis, in situ binding assays and phenotypic characterization under field conditions. Only by clarifying these differences will it be possible to reliably translate regulatory modules from *Arabidopsis* into crop improvement strategies.

### 4.3. Future Application Strategy

Therefore, future research should move beyond homology-based predictions and focus on direct experimental validation. We propose three strategies aimed at translating fundamental knowledge from model plants into practical applications for crop improvement.

Firstly, direct interactions between TFs should be systematically validated. Yeast two-hybrid libraries can be utilized to screen for NAC, MYB, and ERF family members that interact with key WRKY proteins. Integrated omics analyses, combined with transcriptomic data, can further facilitate the construction of regulatory networks and help determine whether NAC, ERF, and MYB promoters are direct targets of WRKY TFs.

**Figure 5:**
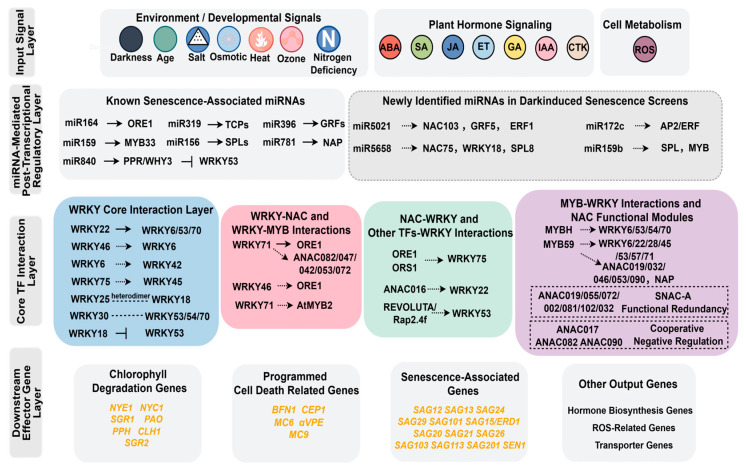
A multi-layer regulatory network of leaf senescence in *Arabidopsis* integrating miRNAs and TFs (WRKY, NAC, MYB). The network consists of four major layers: signal input, miRNA-mediated post-transcriptional regulation, core TF interactions, and downstream target genes. The signal inputs include age, darkness, plant hormones (ABA, SA, JA, ET, GA, IAA, CTK), and ROS. Known senescence-associated miRNAs (miR164, miR319, miR156, miR159, miR396, miR781, miR840) and newly identified miRNAs from dark-induced screens (miR5021, miR5658, miR172c, miR159b) target key TF families. Core TF interactions involve WRKY, NAC, and MYB families, including cross-family interactions and NAC functional modules (SNAC-A redundancy module and ANAC017/082/090 cooperative negative regulation module). Downstream target genes include chlorophyll degradation genes, PCD-related genes, senescence markers, and other output genes.

Secondly, the spatiotemporal dynamics of miRNA-TF modules during senescence should be elucidated (Figure 5). This review identified previously unreported senescence-associated miRNAs, such as *miR5658* and *miR5021*, during a screen for dark-induced senescence, and it also detected changes in the expression of *miR159b* and *miR172c*. Of these, miR159 is known to be involved in regulating the transition from the juvenile to adult stage in *Arabidopsis* [81], whilst miR172 is primarily involved in flowering regulation [82]. Nevertheless, the direct involvement of both factors in leaf senescence has yet to be confirmed. In future studies, the predicted target relationships should be systematically validated using degradome sequencing, 5′RACE, dual-luciferase reporter assays, and transient expression assays. Subsequently, CRISPR/Cas9 technology will be employed to edit conserved miRNA binding sites, followed by the generation of transgenic lines with miRNA overexpression or silencing, in order to elucidate their functional roles in leaf senescence. Conserved miRNA binding sites should be edited using CRISPR/Cas9 technology, and transgenic lines overexpressing or silencing these miRNAs should be generated to clarify their effects on leaf senescence.

Once the molecular interactions have been established, the next step is to elucidate the spatiotemporal dynamics of these miRNA-TF modules during senescence. Single-nucleus RNA sequencing (snRNA-seq) and spatial transcriptomics can be employed to elucidate transcriptional changes across different cell types during the early stages of senescence. When combined with small RNA sequencing and in situ hybridization, these approaches can reveal the spatial expression patterns of miRNAs and their target genes, thereby determining whether senescence possesses cell-type-specific initiation mechanisms. For example, the finding that WRKY41 positively regulates leaf senescence in *Arabidopsis* was based on single-cell nuclear transcriptomics [20]. Although this study did not focus directly on miRNAs, its technical framework and analytical approach can provide valuable insights for research into miRNA-TF modules.

Thirdly, at the application level, future efforts should prioritize assessing the combined effects of these conserved regulatory modules on crop yield, quality and stress tolerance, rather than focusing solely on delaying senescence itself. TF families exhibiting functional redundancy, such as the seven members of the SNAC-A subfamily in *Arabidopsis* and the ANAC017/082/090 module, all demonstrate significant functional redundancy [38]. If similar redundancy exists in crops, single-gene editing is unlikely to achieve the desired improvement. Therefore, future research should utilize multiplex genome editing using CRISPR-Cas9 to construct multi-gene knockout lines in rice, wheat or maize, and systematically evaluate the synergistic roles of redundant members in crop senescence regulation. Concurrently, the practical impact of delayed senescence on yield maintenance and N use efficiency must be assessed under field-relevant conditions such as low N, drought and high temperatures.

Only by combining molecular validation, spatiotemporal analysis and field evaluation can the miRNA-TF regulatory model established in *Arabidopsis* be translated into crop improvement strategies.

## Figures and Tables

**Table 1:** The regulatory role of the WRKY TF in leaf senescence, associated signaling pathways, and representative target genes.

TF	Effects	Signaling Pathway/Regulation	Representative Output Genes	Reference
WRKY1	Promote	SA signaling; induced by N deficiency	*NIA2* ^1^, *NPF5.2* ^1^, *FLC*, *SID2*, *PBS3*, *NIA1*, *NRT3.1*	[14]
WRKY6	Promote	SA signaling; induced by age and pathogen	*PR1* ^1^, *NPR1* ^1^, *SIRK*, *WRKY42*	[15]
WRKY53	Promote	SA and JA signaling; induced by SA, repressed by JA	*WRKY70* ^1^, *SAGs* ^1^, *ESR*	[24,30]
WRKY75	Promote	SA signaling; induced by age and multiple hormones	*SID2*, *CAT2*, *SAG12*, *SAG29*, *WRKY45*	[29,31]
WRKY42	Promote	SA and ROS signaling; induced by senescence	*SIRK* ^1^, *ICS1*, *RbohF*, *SAG13*, *SAG24*, *SAG103*, *PBS3*, *PR1*, *YLS9*	[21]
WRKY55	Promote	SA and ROS signaling; induced by age	*RbohA* ^1^, *SAG29* ^1^, *RbohD*, *ICS1*, *PBS3*, *SAG13*	[26]
WRKY71	Promote	ET signaling; induced by age and ET	*EIN3* ^1^, *SAG29* ^1^, *BFN1* ^1^, *NYE1* ^1^, *SAG13*, *SAG201*, *EIN2*, *ORE1*, *ACS2*	[28]
WRKY22	Promote	Dark signaling; induced by darkness and H_2_O_2_	*SAG12* ^1^, *SAG20* ^1^, *SIRK* ^1^, *WRKY6* ^1^, *WRKY53* ^1^, *WRKY70* ^1^	[17]
WRKY46	Promote	SA signaling; induced by SA	*NPR1* ^1^, *ORE1* ^1^, *NYE1* ^1^, *SEN4* ^1^, *WRKY6*,	[23]
WRKY47	Promote	PCD signaling; induced by age	*SAG29* ^1^, *SEN4* ^1^, *BFN1*, *MC6*, *SAG12*, *SAG13*	[25]
WRKY45	Promote	GA signaling; induced by age and GA	*SAG29* ^1^, *SAG12*, *SAG13*, *SAG113*, *SEN4*	[22]
WRKY41	Promote	Unknown; induced by age	Not specified in the text	[20]
WRKY26	Promote	Unknown; induced by age	Not specified in the text	[18]
WRKY18	Delay	Redox signaling; induced by H_2_O_2_	*WRKY53*	[16]
WRKY54/70	Delay	SA signaling; induced by age	Not specified in the text	[19]
WRKY57	Delay	JA and auxin signaling; induced by JA and IAA	*SAG18* ^1^, *SAG20* ^1^, *SEN4*, *SAG12*	[27]
WRKY30	Complex	SA and ROS signaling; induced by age, SA, H_2_O_2_, and ozone	Not specified in the text	[19]
WRKY25	Complex	Redox signaling; induced by H_2_O_2_	*WRKY53*	[16]

^1^ Expression validation only (RNA-Seq/microarray/qRT-PCR). No superscript: validated by direct interaction assays (ChIP-qPCR/EMSA/dual luciferase/yeast one-hybrid).

**Table 2:** The regulatory role of the NAC TF in leaf senescence, associated signaling pathways, and representative target genes.

TF	Effects	Signaling Pathway/Regulation	Representative Output Genes	Reference
ANAC002 (ATAF1)	Promote	ABA and H_2_O_2_ signaling; induced by age, ABA, H_2_O_2_, and darkness	*ORE1*, *GLK1*, *NCED3*, *ABCG40*, *SAG12* ^1^, *SAG13* ^1^, *BFN1* ^1^, *SWEET15* ^1^	[39]
ANAC059 (ORS1)	Promote	H_2_O_2_ and salt stress signaling; induced by H_2_O_2_, salt, and age	*WRKY75* ^1^, *SEN1* ^1^, *DIN11* ^1^, *YLS9* ^1^, *ALD1* ^1^, *FMO1* ^1^, *PDF1.4* ^1^, *PDR12* ^1^, *AOX1D* ^1^	[35]
ANAC032	Promote	ROS and auxin signaling; induced by age, darkness, H_2_O_2_, ABA, MeJA, salt, and osmotic stress	*AtNYE1* ^1^, *PPH* ^1^, *SAG12* ^1^, *SAG113* ^1^, *SAUR36* ^1^	[41]
ANAC029 (AtNAP)	Promote	Age-dependent senescence signaling; induced by age and PCD	*SAG12* ^1^, *SAG13* ^1^, *RBCS* ^1^	[34]
ANAC102/019/055/081/032	Promote	ABA signaling; induced by ABA and age	*SEN4* ^1^, *SAG12* ^1^, *SAG13* ^1^, *SAG15/ERD1* ^1^, *SAG26* ^1^, *SAG201* ^1^, *SAG113* ^1^, *ATH7* ^1^, *ATH8* ^1^, *GLY14* ^1^, *GLY17* ^1^	[38]
ANAC016	Promote	ABA, MeJA, salt, and H_2_O_2_ signaling; induced by age, darkness, ABA, MeJA, salt, and H_2_O_2_	*NAP*, *ORS1*, *JUB1* ^1^, *VNI2* ^1^, *ORE1* ^1^, *SGR1* ^1^, *PPH* ^1^, *NYC1* ^1^, *WRKY22* ^1^	[37]
ANAC087	Promote	Age-dependent senescence signaling; induced by age	*NYE1*, *BFN1*, *RbohD*, *SAG13*, *SAG15*, *CEP1*, *MC9*, *PAO* ^1^, *PPH* ^1^, *αVPE* ^1^	[46]
ANAC092 (ORE1)	Promote	Salt and ethylene signaling; induced by age, salt, darkness, and wounding	*ANAC041* ^1^, *ANAC054* ^1^, *ANAC083* ^1^, *SAG12* ^1^, *BFN1* ^1^, *NYE1* ^1^, *WRKY75* ^1^, *ALD1* ^1^, *FMO1* ^1^, *PDF1.4* ^1^, *PDR12* ^1^, *AOX1D*	[45]
ANAC072 (RD26)	Promote	Age and dark signaling; induced by age and darkness	*NYE1*	[40]
ANAC046	Promote	JA and ET signaling; induced by age and darkness	*NYC1*, *SGR1*, *SGR2*, *PAO*, *PPH* ^1^, *CLH1* ^1^, *SAG12* ^1^, *SAG13* ^1^	[42]
ANAC082	Delay	ROS signaling; induced by age	*SEN4* ^1^, *SAG12* ^1^	[43]
ANAC090	Delay	SA signaling; induced by age	*SEN4* ^1^, *SAG12* ^1^, *PR1*, *EDS5*, *SIRK*, *ICS1*, *ANAC042*, *ANAC046*, *ANAC047*, *ANAC053*, *ANAC081*, *ANAC087*, *AT3G12910*	[43]
ANAC017	Delay	ROS signaling; induced by age	*SEN4* ^1^, *SAG12* ^1^, *DUF239* ^1^, *BCB* ^1^, *MSRB8* ^1^, *At2g21640* ^1^	[43]
ANAC083 (VNI2)	Delay	ABA-dependent salinity signaling; induced by ABA and salt	*SAG12* ^1^, *COR15a*, *COR15b*, *RD29A*, *RD29B*	[44]
ANAC042 (JUB1)	Delay	H_2_O_2_ signaling; induced by H_2_O_2_, salinity, heat, and oxidative stress	*DREB2A*, *HSP101* ^1^, *HSP17.6* ^1^, *HSP70* ^1^, *GSTU10* ^1^, *SAG12* ^1^, *DFR* ^1^, *PAP1* ^1^	[36]
ANAC075	Delay	ABA, ET, MeJA, SA, H_2_O_2_, and salt signaling; induced by age and multiple hormones	*CAT2*	[47]

^1^ Expression validation only (RNA-Seq/microarray/qRT-PCR). No superscript: validated by direct interaction assays (ChIP-qPCR/EMSA/dual luciferase/yeast one-hybrid).

**Table 3:** The regulatory role of the MYB and other TF families in leaf senescence, associated signaling pathways, and representative target genes.

TF	Effects	Signaling Pathway/Regulation	Representative Output Genes	Reference
AtMYBL	Promote	ABA and salt signaling; induced by age, ABA, and salt	*SAG12* ^1^, *SRG1* ^1^, *RBCS1* ^1^, *LHB1B2* ^1^, *RD29A* ^1^, *RD29B* ^1^	[49]
GL1	Promote	Dark-induced senescence signaling; induced by darkness	*SAG12* ^1^, *SAG13* ^1^, *SEN1* ^1^, *RbcS1A* ^1^, *CAB1* ^1^, *CAB2* ^1^, *PAL1* ^1^	[52]
MYBH	Promote	Auxin signaling; induced by age and darkness	*DFL1/GH3.6*, *DFL2/GH3.10*, *WRKY6* ^1^, *WRKY53* ^1^, *WRKY54* ^1^, *WRKY70* ^1^, *SAG12* ^1^, *CAB2* ^1^, *SAUR36* ^1^, *IAA29* ^1^, *BBX25* ^1^, *BBX27* ^1^, *RPT2* ^1^	[50]
AtMYB2	Promote	CTK signaling; induced by age	*IPT1* ^1^, *IPT4* ^1^, *IPT5* ^1^, *IPT6* ^1^, *IPT8* ^1^	[51]
Rap2.4f	Promote	Abiotic stress signaling; induced by salt, mannitol, and darkness	*SAG12* ^1^, *SAG13* ^1^, *SAG24* ^1^, *SAG101* ^1^, *ANAC083* ^1^, *WRKY53* ^1^, *SIRK* ^1^, *LHCA1* ^1^	[60]
RAV1	Promote	ET and JA signaling; induced by ACC and MJ	*CAB2* ^1^, *SEN4* ^1^, *SAG12* ^1^, *SAG13* ^1^, *SAG24* ^1^, *ERF* ^1^, *PDF* ^1^	[59]
REVOLUTA	Promote	Redox signaling; induced by H_2_O_2_	*WRKY53*, *HAT3*, *ZFP8*, *AT1G49200*, *AT1G74940*, *IDD11*, *AT5G47180*, *SAG12* ^1^, *SAG13* ^1^	[61]
CDF4	Promote	ABA and ROS signaling; induced by ABA, SA, H_2_O_2_, and darkness	*NCED2*, *NCED3*, *CAT2*, *PGAZAT*, *SAG12* ^1^, *SAG13* ^1^	[57]
AtFOX1	Promote	ABA signaling; induced by ABA	*TGA7*, *ABF2*, *ABF3*, *SAG12* ^1^, *RD29B* ^1^, *RAB18* ^1^, *PP2CA* ^1^	[58]
ORE15	Delay	GRF/GIF regulatory module signaling; induced by developmental age	*GRF1*, *GRF4*, *GIF1/AN3* ^1^, *GRF5* ^1^, *ANT* ^1^, *CYCD3;1* ^1^, *miR396a* ^1^, *ORE1* ^1^, *SEN4* ^1^, *SAG12* ^1^	[56]
MYB59	Delay	Leaf senescence signaling; induced by SA- and JA-mediated senescence	*SAG18*, *ICS1*, *PAL2*, *LOX2*, *SEN4* ^1^, *SAG12* ^1^, *SAG13* ^1^, *SAG20* ^1^, *SAG21* ^1^, *SAG24* ^1^, *SAG29* ^1^, *SAG101* ^1^, *NYE1* ^1^, *NYE2* ^1^, *NAC019* ^1^, *NAC032* ^1^, *NAC046* ^1^, *NAC053* ^1^, *NAC090* ^1^, *NAP* ^1^, *WRKY6* ^1^, *WRKY22* ^1^, *WRKY28* ^1^, *WRKY45* ^1^, *WRKY53* ^1^, *WRKY57* ^1^, *WRKY71* ^1^	[54]
MYB47	Delay	JA signaling; induced by JA	*CYP94B3*, *CYP94C1*, *VSP1*^1^, *PDF1.2*^1^, *THI2.1*^1^, *MYC2*^1^, *SAG12*^1^	[55]
MYBR1 (MYB44)	Delay	ABA signaling; induced by ABA and ABA-dependent salt stress	*PYL8*, *MYBR2*, *RD29A*, *COR15A/B* ^1^, *RD29B* ^1^, *KIN1/2* ^1^, *RAB18* ^1^, *DREB2A* ^1^, *P5CS1* ^1^, *SAG12* ^1^, *SAG29* ^1^, *SEN4* ^1^	[53]

^1^ Expression validation only (RNA-Seq/microarray/qRT-PCR). No superscript: validated by direct interaction assays (ChIP-qPCR/EMSA/dual luciferase/yeast one-hybrid).

## Data Availability

No new data were created or analyzed in this study. Data sharing is not applicable to this article.

## Abbreviations

The following abbreviations are used in this manuscript:

TF	Transcription Factor
miRNA	microRNA
SAGs	Senescence-Associated Genes
N	Nitrogen
SA	Salicylic Acid
JA	Jasmonic Acid
ET	Ethylene
ABA	Abscisic Acid
PCD	Programmed Cell Death
ROS	Reactive Oxygen Species
H_2_O_2_	Hydrogen Peroxide
GA	Gibberellin
IAA	Indole-3-Acetic Acid
*NIA2*	*NITRATE REDUCTASE 2*
*NPF5.2*	*NRT1/PTR FAMILY 5.2*
*FLC*	*FLOWERING LOCUS C*
*SID2*	*SA INDUCTION DEFICIENT 2*
*PBS3*	*AVRPPHB SUSCEPTIBLE 3*
*NIA1*	*NITRATE REDUCTASE 1*
*NRT3.1*	*NITRATE TRANSPORTER 3.1*
*PR1*	*PATHOGENESIS-RELATED PROTEIN 1*
*NPR1*	*NONEXPRESSER OF PR GENES 1*
*SIRK*	*SENESCENCE-INDUCED RECEPTOR-LIKE KINASE*
*CAT**2*	*CATALASE 2*
*ICS1*	*ISOCHORISMATE SYNTHASE 1*
*RbohF*	*RESPIRATORY BURST OXIDASE HOMOLOG F*
*YLS9*	*YELLOW-LEAF-SPECIFIC GENE 9*
*RbohA*	*RESPIRATORY BURST OXIDASE HOMOLOG A*
*EIN3*	*ETHYLENE INSENSITIVE 3*
*BFN1*	*BIFUNCTIONAL NUCLEASE 1*
*NYE1*	*NON-YELLOWING 1*
*EIN2*	*ETHYLENE INSENSITIVE 2*
*ORE1*	*ORESARA 1*
*ACS2*	*ACC SYNTHASE 2*
*SEN4*	*SENESCENCE-ASSOCIATED GENE 4*
*MC6*	*METACASPASE 6*
RNA-Seq	RNA Sequencing
ChIP-qPCR	Chromatin Immunoprecipitation quantitative Polymerase Chain Reaction
EMSA	Electrophoretic Mobility Shift Assay
qRT-PCR	quantitative Real-Time Polymerase Chain Reaction
*ORS1*	*ORGAN SIZE-LIKE PROTEIN*
*JUB1*	*JUNGBRUNNEN 1*
MeJA	Methyl Jasmonate
*VNI2*	*VND-INTERACTING 2*
*GLK1*	*GOLDEN2-LIKE 1*
*NCED3*	*NINE-CIS-EPOXYCAROTENOID DIOXYGENASE 3*
*ABCG40*	*ABC TRANSPORTER G FAMILY MEMBER 40*
*SWEET15*	*SUGARS WILL EVENTUALLY BE EXPORTED TRANSPORTER 15*
*SEN1*	*SENESCENCE-ASSOCIATED GENE 1*
*DIN11*	*DARK-INDUCIBLE GENE 11*
*ALD1*	*AGD2-LIKE DEFENSE RESPONSE PROTEIN 1*
*FMO1*	*FLAVIN-DEPENDENT MONOOXYGENASE 1*
*PDF1.4*	*PLANT DEFENSIN 1.4*
*PDR12*	*PLEIOTROPIC DRUG RESISTANCE 12*
*AOX1D*	*ALTERNATIVE OXIDASE 1D*
*AtNYE1*	*ARABIDOPSIS NON-YELLOWING 1*
*PPH*	*PHEOPHYTINASE*
*SAUR36*	*SMALL AUXIN UP RNA 36*
*RBCS*	*RUBISCO SMALL SUBUNIT*
*ERD1*	*EARLY RESPONSIVE TO DEHYDRATION 1*
*ATH7*	*ARABIDOPSIS THALIANA METALLOTHIONEIN 7*
*ATH8*	*ARABIDOPSIS THALIANA METALLOTHIONEIN 8*
*GLY14*	*GLYOXALASE 14*
*GLY17*	*GLYOXALASE 17*
*NAP*	*NAC-LIKE*, *ACTIVATED BY AP3/PI*
*SGR1*	*STAY-GREEN 1*
*NYC1*	*NON-YELLOW COLORING 1*
*RbohD*	*RESPIRATORY BURST OXIDASE HOMOLOG D*
*CEP1*	*CYSTEINE ENDOPEPTIDASE 1*
*MC**9*	*METACASPASE 9*
*PAO*	*PHEOPHORBIDE A OXYGENASE*
*α**VPE*	*ALPHA VACUOLAR PROCESSING ENZYME*
*CLH1*	*CHLOROPHYLLASE 1*
*EDS5*	*ENHANCED DISEASE SUSCEPTIBILITY 5*
*DUF239*	*DOMAIN OF UNKNOWN FUNCTION 239*
*BCB*	*BLUE COPPER-BINDING PROTEIN*
*MSRB8*	*METHIONINE SULFOXIDE REDUCTASE B8*
*COR15a*	*COLD-REGULATED 15a*
*COR15b*	*COLD-REGULATED 15b*
*RD29A*	*RESPONSIVE TO DEHYDRATION 29A*
*RD29B*	*RESPONSIVE TO DEHYDRATION 29B*
*DREB2A*	*DEHYDRATION-RESPONSIVE ELEMENT-BINDING PROTEIN 2A*
*HSP*	*HEAT SHOCK PROTEIN*
*GSTU10*	*GLUTATHIONE S-TRANSFERASE TAU 10*
*DFR*	*DIHYDROFLAVONOL REDUCTASE*
*PAP1*	*PRODUCTION OF ANTHOCYANIN PIGMENT 1*
*COR*	*COLD-REGULATED*
*RD*	*RESPONSIVE TO DEHYDRATION*
CTK	Cytokinin
GL1	GLABRA 1
*PAL2*	*PHE AMMONIA LYASE 2*
*LOX2*	*LIPOXYGENASE 2*
*CYP94B3*	*CYTOCHROME P450 94B3*
ORE15	ORESARA 15
PLATZ	PLANT A/T-RICH SEQUENCE- AND ZINC-BINDING PROTEIN
*GIF1*	*GRF-INTERACTING FACTOR 1*
*AN3*	*ANGUSTIFOLIA 3*
*GRF5*	*GROWTH-REGULATING FACTOR 5*
CDF4	CYCLING DOF FACTOR 4
DOF	DNA BINDING WITH ONE FINGER
*NCED2*	*NINE-CIS-EPOXYCAROTENOID DIOXYGENASE 2*
FOX	FORKHEAD BOX
ACC	1-aminocyclopropane-1-carboxylic acid
RAV1	RELATED TO ABI3/VP1 1
*RbcS1A*	*RUBISCO SMALL SUBUNIT 1A*
*CAB1*	*CHLOROPHYLL A/B BINDING PROTEIN 1*
*CAB**2*	*CHLOROPHYLL A/B BINDING PROTEIN 2*
*PAL1*	*PHE AMMONIA LYASE 1*
*DFL1*	*DWARF IN LIGHT 1*
*GH3.6*	*GRETCHEN HAGEN 3.6*
*DFL2*	*DWARF IN LIGHT 2*
*GH3.10*	*GRETCHEN HAGEN 3.10*
*IAA29*	*INDOLE-3-ACETIC ACID INDUCIBLE 29*
*BBX25*	*B-BOX DOMAIN PROTEIN 25*
*RPT2*	*ROOT PHOTOTROPISM 2*
*IPT1*	*ISOPENTENYLTRANSFERASE 1*
*LHCA1*	*LIGHT-HARVESTING COMPLEX I SUBUNIT A1*
*ERF1*	*ETHYLENE RESPONSE FACTOR 1*
*HAT3*	*HOMEODOMAIN-LEUCINE ZIPPER PROTEIN 3*
*ZFP8*	*ZINC FINGER PROTEIN 8*
*IDD11*	*INDETERMINATE DOMAIN PROTEIN 11*
*TGA7*	*TGACG SEQUENCE-SPECIFIC BINDING PROTEIN 7*
*ABF2*	*ABSCISIC ACID-RESPONSIVE ELEMENT BINDING FACTOR 2*
*RAB18*	*RESPONSIVE TO ABA 18*
*PP2CA*	*PROTEIN PHOSPHATASE 2CA*
*ANT*	*AINTEGUMENTA*
*CYCD3;1*	*CYCLIN D3;1*
*VSP1*	*VEGETATIVE STORAGE PROTEIN 1*
*THI2.1*	*THIONIN 2.1*
*MYC2*	*MYC TRANSCRIPTION FACTOR 2*
*PYL8*	*PYRABACTIN RESISTANCE 1-LIKE 8*
*MYBR2*	*MYB TRANSCRIPTION FACTOR REPRESSOR 2*
*KIN1*	*COLD-INDUCIBLE PROTEIN 1*
*P5CS1*	*DELTA-1-PYRROLINE-5-CARBOXYLATE SYNTHASE 1*
TCP	TEOSINTE BRANCHED/CYCLOIDEA/PCF
SPL	SQUAMOSA PROMOTER BINDING PROTEIN-LIKE
*HST*	*HASTY*
DCL1	DICER-LIKE 1
RAN1	RAS-RELATED NUCLEAR PROTEIN 1
*PPR*	*PENTATRICOPEPTIDE REPEAT*
*WHY3*	*WHIRLY 3*
ABI5	ABA INSENSITIVE 5
*UBQ6*	*Ubiquitin Extension Protein 6*
AP2	APETALA2
VQ8	VQ MOTIF-CONTAINING PROTEIN 8
bZIP	basic Leucine Zipper
*ABF**1*	*ABSCISIC ACID-RESPONSIVE ELEMENT BINDING FACTOR 1*
*PAL5*	*PHENYLALANINE AMMONIA LYASE 5*
*CAD14*	*CINNAMYL ALCOHOL DEHYDROGENASE 14*
CRISPR	Clustered Regularly Interspaced Short Palindromic Repeats
Cas9	CRISPR-associated protein 9
